# Water Oxidation and Degradation Mechanisms of BiVO_4_ Photoanodes in Bicarbonate Electrolytes

**DOI:** 10.1021/acscatal.5c03025

**Published:** 2025-07-16

**Authors:** Guanda Zhou, Clara C. Aletsee, Anna Lemperle, Tim Rieth, Lucia Mengel, Jianyong Gao, Martin Tschurl, Ueli Heiz, Ian D. Sharp

**Affiliations:** a Walter Schottky Institute, 9184 Technical University of Munich, Garching 85748, Germany; b Physics Department, TUM School of Natural Sciences, Technical University of Munich, Garching 85748, Germany; c Chair of Physical Chemistry, TUM School of Natural Sciences and Catalysis Research Center, Technical University of Munich, Garching 85748, Germany

**Keywords:** bismuth vanadate, bicarbonate electrolytes, hydrogen peroxide evolution reaction, stability, photoanodes

## Abstract

The photoelectrochemical hydrogen peroxide evolution reaction (HPER) has attracted increasing attention as an environmentally friendly approach to generate a commercially and industrially valuable water oxidation product. BiVO_4_ photoanodes operated in bicarbonate-containing electrolytes have been shown to offer remarkable performance characteristics for HPER, with HCO_3_^–^ serving as a reaction mediator. However, the factors affecting the stability of both the semiconductor photoanode and the aqueous electrolyte remain poorly understood. Here, we investigated BiVO_4_ photoanodes to quantitatively assess the roles of electrolyte composition, bias potential, and illumination on competitive reaction pathways associated with HPER, oxygen evolution reaction, and photocorrosion. Our results confirm that HCO_3_^–^ serves as a highly efficient mediator, leading to rapid hole extraction and near complete suppression of interfacial recombination on BiVO_4_. In addition, these favorable hole transfer kinetics significantly decrease the rate of photocorrosion, leading to dramatically enhanced stability compared to bicarbonate-free electrolytes. While the elevated pH of unbuffered bicarbonate electrolyte leads to gradual chemical attack of BiVO_4_, the stability is greatly enhanced in near-neutral buffered bicarbonate electrolytes. Finally, we confirm that HCO_3_^–^ is regenerated during the photoanodic reaction, though pH swings during operation in an unbuffered electrolyte can lead to electrolyte instabilities. Overall, we find that BiVO_4_ photoanodes operating in buffered bicarbonate-containing solutions exhibit significantly enhanced stability and can efficiently drive water oxidation reactions, including HPER, thus providing a route to robust production of high value oxidation products.

## Graphical abstract

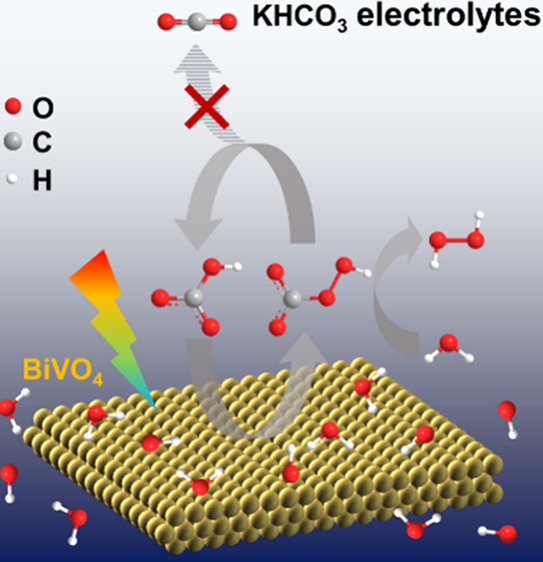

## 1 Introduction

Bismuth vanadate (BiVO_4_) has recently emerged as a promising semiconductor photoanode for photoelectrochemical (PEC) water oxidation to hydrogen peroxide (H_2_O_2_),^1–10^ an oxidizing agent that has a wide range of applications in the chemical, manufacturing, and medical sectors.^11,12^ Compared to the anthraquinone method that is currently used for industrial H_2_O_2_ synthesis, the PEC route offers considerable advantages as a sustainable, renewable, and environmentally friendly alternative.^13,14^ While BiVO_4_ has been more commonly investigated as a photoanode for driving the oxygen evolution reaction (OER) in integrated solar water splitting devices,^15–18^ molecular oxygen offers little value and is typically vented to the atmosphere. In contrast, H_2_O_2_ is a significantly more valuable chemical product that can provide added benefit from the oxidation half reaction. Furthermore, it has been shown that operation of BiVO_4_ in carbonate-containing electrolytes leads to dramatically enhanced Faradaic efficiencies for the hydrogen peroxide evolution reaction (HPER),^19,20^ which could enable synergistic coupling of BiVO_4_ photoanodes with (photo)­cathodes that drive CO_2_ reduction reactions (CO2RR) in the same electrolyte environment.

In general, PEC OER and HPER can proceed according to the respective reactions:

$$2H_{2}O+4h^{+}\to O_{2}+4H^{+}(E^{{}^{\circ}}=1.23\;V)$$ 1a

$$2H_{2}O+2h^{+}\to H_{2}O_{2}+2H^{+}(E^{{}^{\circ}}=1.76\;V)$$ 1b

Although the two-hole HPER is kinetically faster than the four-hole OER, the reaction is thermodynamically unfavorable, as the reversible potential for H_2_O_2_ generation (1.76 V vs RHE) is higher than that for O_2_ generation (1.23 V vs RHE).^21^ Therefore, H_2_O_2_ selectivities and yields have been reported to be extremely low in most electrolytes, including phosphate buffer (KPi), borate buffer (KBi), and sodium sulfate solution (Na_2_SO_4_).^22,23^ Nevertheless, significantly higher H_2_O_2_ yields have been obtained in bicarbonate-containing electrolytes.^20,24^ This important observation has been attributed to a different chemical reaction pathway, in which the bicarbonate anion participates as a reaction mediator, accepting two holes from the photoelectrode to generate peroxymonocarbonate (HCO_4_^–^) that then homogeneously oxidizes water to release H_2_O_2_ and regenerate bicarbonate according to^25,26^

$${{\mathrm{HCO}}_{3}}^{-}+H_{2}O+2h^{+}\to {{\mathrm{HCO}}_{4}}^{-}+2H^{+}$$ 2a

$${{\mathrm{HCO}}_{4}}^{-}+H_{2}O⇌H_{2}O_{2}+{{\mathrm{HCO}}_{3}}^{-}$$ 2b

In this way, bicarbonate effectively serves as a catalyst for HPER, accelerating the reaction rate relative to OER and reducing the onset potential for photocurrent generation.

Among photoanodes, BiVO_4_ offers best-in-class selectivities for PEC HPER, which has been attributed to a combination of its energetically deep valence band position, moderate bandgap (∼2.5 eV) for solar energy harvesting, and poor native activity for OER. However, BiVO_4_ is also known to suffer from photocorrosion under aqueous conditions.^27^ While these instabilities can be mitigated through the application of catalytic coatings,^28–31^ HPER typically proceeds on the bare surface of the semiconductor, which is directly exposed to the bicarbonate-containing electrolyte. Quantitative studies of Bi and V leaching in various electrolytes have been previously reported,^27,32–34^ indicating that the material degrades almost stoichiometrically in KPi buffer^27^ and predominantly via V-dissolution in KBi buffer.^34^ However, much less is known about its (photo)­electrochemical corrosion characteristics in bicarbonate-based electrolytes. Thus, a critical assessment of the factors affecting stability under HPER-relevant conditions is needed. Moreover, since bicarbonate is believed to mediate HPER, characterizing the stability of not just BiVO_4_ but also the electrolyte is a key requirement for establishing whether the complete photoanodic reaction process is robust. While most studies have concluded that the regenerative reaction pathway described by eq 2 is most likely responsible for HPER on BiVO_4_, it has also been speculated that HCO_3_^–^ anions can serve as a nonregenerative hole scavenger that can be oxidized and release CO_2_, as well as O_2_ or H_2_O_2_, according to^20^

$${{2\mathrm{HCO}}_{3}}^{-}+{4h}^{+}\to {2\mathrm{CO}}_{2}+O_{2}+{2H}^{+}$$ 3a

$${{\mathrm{HCO}}_{3}}^{-}+H_{2}O+2h^{+}\to {\mathrm{CO}}_{2}+H_{2}O_{2}+H^{+}$$ 3b

Although CO_2_ could be redissolved through its aqueous equilibration reaction, its loss to the gas phase in open systems would represent a decomposition of the electrolyte and considerably alter the total energy efficiency for HPER. However, most studies of HPER with BiVO_4_ have focused on H_2_O_2_ quantification, with only some measuring evolved O_2_ and, to the best of our knowledge, none analyzing CO_2_ loss.^1,4–6,8–10,20,21,23,35–37^

In the present work, we provide a detailed investigation of the role of HCO_3_^–^ on both the operation and stability of BiVO_4_ photoanodes. Our results confirm that HCO_3_^–^ anions promote PEC responses by rapidly extracting photogenerated holes, leading to a considerable increase in the interfacial charge carrier injection efficiency relative to a bicarbonate-free KPi electrolyte. Importantly, photocarrier recombination at the semiconductor/electrolyte interface can be nearly completely suppressed in the presence of HCO_3_^–^, resulting in a dramatic decrease in the rate of photocorrosion compared to KPi. Nevertheless, comparison of unbuffered and buffered KHCO_3_ electrolytes reveals very different chemical stabilities of BiVO_4_ photoanodes. In particular, using inductively coupled plasma mass spectroscopy (ICP-MS), we find considerable leaching of both Bi and V into the electrolyte for the case of unbuffered KHCO_3_, which we attribute to chemical attack under mildly alkaline conditions. In contrast, buffered KHCO_3_ enables efficient and stable operation of BiVO_4_ with minimal corrosion. Finally, to assess whether HCO_3_^–^ is regenerated (eq 2) or consumed (eq 3) during sustained operation of BiVO_4_ photoanodes, we quantified O_2_ and H_2_O_2_ generation from both unbuffered and buffered systems, as well as CO_2_ generation under unbuffered conditions. Although CO_2_ is released, we find that this is not a consequence of photochemical decomposition of the electrolyte, but rather is primarily due to the reduced pH of the anolyte that inevitably occurs during continuous photoanodic operation under unbuffered conditions. Thus, we conclude that buffered bicarbonate-containing electrolytes are extremely beneficial for achieving both efficient and stable operation of BiVO_4_ photoanodes, allowing the chemical and photochemical instabilities of this material to be overcome. At the same time, such conditions enable sustainable generation of H_2_O_2_ as a valuable oxidation product and are compatible with the environments needed for driving complementary (photo)­cathodic CO_2_ reduction reactions in integrated systems.

## 2 Methods

### 2.1 BiVO_4_ Synthesis

Prior to sample fabrication, fluorine-doped tin oxide (FTO, Sigma-Aldrich, ∼ 7 Ω/sq) and fused silica (Siegert Wafer, UV grade) substrates were cleaned with acetone (VWR, 99%), isopropanol (VWR, > 99%), and deionized water (DI water, 18 MΩ cm). The substrates were subsequently dried under flowing N_2_ and annealed at 500 °C for 30 min to improve the wettability of the surface.

BiVO_4_ photoanodes were prepared by spin coating and metal–organic decomposition based on a previously reported method.^38,39^ Specifically, 0.379 g Bi­(NO_3_)_3_·5H_2_O (0.2 M, Sigma-Aldrich, 98%) and 0.2076 g OV­(C_5_H_7_O_2_)_2_ (0.03 M, Sigma-Aldrich, 98%) were dissolved in 4 and 26 mL acetylacetone (Sigma-Aldrich, > 99.5%), respectively. The two solutions were then mixed and subjected to ultrasonication for 5 min. To remove large particles, the mixture was filtered with PET filters (Carl Roth, 0.2 μm pore size). To form thin films, 0.5 mL of the resulting solution was spin coated onto FTO or fused silica substrates at a rotation speed of 1000 rpm, followed by calcination at 500 °C for 10 min in a muffle furnace (Nabertherm LT 3/12) to evaporate the solvent and crystallize the BiVO_4_. This coating and annealing process was repeated 12 times, after which the samples were annealed at 500 °C for 2 h.

### 2.2 Materials Characterization

UV–vis absorption spectra of BiVO_4_ were obtained using an Agilent Cary 5000 spectrometer in the double-beam configuration. To determine the bandgap of as-prepared BiVO_4_, Tauc analysis was carried out by fitting the data with the following equation:^40^

$${\left(\alpha h\upsilon \right)}^{n}=B(h\upsilon -E_{g})$$ 4

where α is the absorption coefficient, *B* is a constant, *E*_*g*_ is the bandgap of BiVO_4_, and *n* has a value of 0.5 for indirect and of 2 for direct bandgap semiconductors. Considering the indirect bandgap of BiVO_4_,^40^ a value of *n* = 0.5 was used for fitting.

Grazing incidence X-ray diffraction (GIXRD) patterns were collected on a Rikagu SmartLAB X-ray diffractometer. The incidence angle was fixed at 0.5° and the 2*θ* range was scanned between 10 and 60° with a scan speed of 2°/min. Surface morphologies and film thicknesses were characterized using a Carl Zeiss NVision 40 scanning electron microscope (SEM) with an electron acceleration of 5 kV. In addition, surface morphologies and quantitative rms surface roughness values (R_q_) were characterized using atomic force microscopy (AFM, Bruker Dimension Icon XR) in peak force tapping mode.

### 2.3 Photoelectrochemical Measurements

PEC measurements were performed using a commercial two-compartment electrochemical cell (Gauss Union), with each compartment having a volume of 118 mL and separated by a Fumasep FAA-75/130 anion exchange membrane. The measurements were conducted under open-air conditions and under continuous stirring, unless otherwise noted. The PEC performance characteristics were recorded using a Biologic SP-300 potentiostat in a three-electrode configuration, including the BiVO_4_ photoanode as working electrode, a 3 M Ag/AgCl reference electrode, and a Pt counter electrode. An Asahi HAL-320 solar simulator with a light intensity of 100 mW/cm^2^ (1 Sun, AM1.5G condition) was employed as the light source. The measured electrochemical potentials vs the Ag/AgCl reference electrode (*E*_Ag/AgCl_) were converted to the reversible hydrogen electrode (*E*_RHE_) scale using the Nernst equation, as follows:^41^

$$E_{\mathrm{RHE}}=E_{\mathrm{Ag}/\mathrm{AgCl}}+0.21V+\left(0.059\mathrm{pH}\right)$$ 5

Prior to all PEC measurements, five voltammetry cycles were conducted to ensure stable measurement conditions. To assess the maximum light-limited photocurrent density for the films used in this study, we considered that the wavelength-dependent absorbed light power (*P*_abs_(λ)) for a given absorbance (*A*(λ)) is given by^42^

$$P_{\mathrm{abs}}\left(\lambda \right)=P_{0}\left(\lambda \right)\left(1-10^{-A\left(\lambda \right)}\right)$$ 6

where *P*_0_(λ) is the wavelength-dependent power of the ASTM G173 AM1.5G solar spectrum.^43^ The value of *A*(λ), which depends on the bandgap and film thickness, was measured by UV–vis spectroscopy, as described above. In the absence of recombination losses during photon-to-current conversion, the maximum photocurrent density (*J*_abs_) can be calculated by integrating the absorbed photon flux according to^42^

$$J_{\mathrm{abs}}=\int_{\lambda_{1}}^{\lambda_{2}}\frac{\lambda }{1240}P_{\mathrm{abs}}\left(\lambda \right)d\lambda$$ 7

In the present case, the calculated *J*_abs_ of BiVO_4_ is 2.88 mA/cm^2^, which is less than the light-limited saturation current density for the ∼ 2.5 eV bandgap semiconductor due to the thickness of the films (∼60 nm), as well as reflection and scattering losses at the front surface.

### 2.4 Transient Photocurrent Measurements

The approach to transient photocurrent measurements has been previously reported.^41^ In brief, the BiVO_4_ photoanode was held at specified electrochemical potentials under continuous illumination (100 mW/cm^2^, Asahi HAL-320), and transients were recorded upon illumination with a chopped ∼ 10 mW/cm^2^ 365 nm LED light source (Thorlabs M365LP1), cycled with 200 ms on and 100 ms off times. The applied potentials were adjusted from 0.2 to 1.0 V vs the open circle potential (*E*_oc_), and the LED illumination-induced photocurrent transients were recorded using a Biologic SP300 potentiostat. Similar experiments were also carried out upon specific potentials under 20 mW/cm^2^ 365 nm LED illumination only.

### 2.5 Inductively Coupled Plasma Mass Spectrometry (ICP-MS)

The stabilities of BiVO_4_ photoanodes in 0.5 M KPi buffer (pH = 6.8, Sigma-Aldrich), 0.5 M unbuffered KHCO_3_ electrolyte (pH = 8.5, Sigma-Alrich), and 0.5 M KHCO_3_ buffer with continuous CO_2_ (Linde, 99.999%) bubbling at a rate of 50 sccm (pH = 7.5) were characterized using ICP-MS to quantify the dissolved ion concentrations following chronoamperometric testing. To compare the influence of illumination, bias, and pH on the stabilities of BiVO_4_ photoanodes, four reaction conditions were employed in each electrolyte, including dark measurements at *E*_oc_ (*E*_*oc*_^*dark*^) and at 1.23 V_RHE_ (*E*_1.23 *V*_^*dark*^), as well as illuminated measurements at *E*_oc_ (*E*_*oc*_^*light*^) and 1.23 V_RHE_ (*E*_1.23 *V*_^*light*^). Although the reversible potential for water oxidation to H_2_O_2_ lies at a higher potential (1.76 V_RHE_), we selected 1.23 V_RHE_ as the operating point to avoid contributions from dark electrochemical processes. In addition, we note that both OER and HPER can be driven at 1.23 V_RHE_ under illumination due to the photovoltage generated by BiVO_4_ photoanodes. Fresh BiVO_4_ photoanodes were used for each measurement condition, with 1 mL of electrolyte collected after 2 h chronoamperometry experiments. The Bi and V concentrations were determined using an Agilent 7900 ICP-MS operated in He collision mode and high matrix mode with Ar dilution. Prior to the measurements, the collected liquid samples were prediluted at a 1:20 ratio with 2% HNO_3_ (prepared from 70% HNO_3_ ROTIPURAN Supra, < 1 ppb trace metals and DI water). Bi and V calibration series were freshly prepared from certified multielement standards (TraceCERT, Post-transition Metal Mix, and Transition Metal Mix 1, respectively) and the same 2% HNO_3_. During the ICP-MS measurement, an internal standard containing rare earth elements was added to verify measurement stability across samples. Assuming uniform film degradation, the etching rate, *Ṙ*, was then calculated as follows:

$$Ṙ=\frac{CV}{\rho A}\frac{1}{t}$$ 8

where *C* is the mass concentration of dissolved BiVO_4_ measured by ICP-MS, *V* is the volume of the electrolyte, ρ is the density of BiVO_4_ (6.1 g/cm^3^), *A* is the exposed area of the BiVO_4_ photoanode, and *t* is the chronoamperometric reaction time at which the liquid sample was collected. The experiment under the same conditions was repeated twice to calculate the mean and error bars.

### 2.6 Spectroscopic Characterization of Degraded Samples

According to the Beer–Lambert law, the absorption signal (*A*) recorded by the spectrometer can be written as:^3^

$$A=\log\;\frac{I}{I_{0}}=-\alpha d$$ 9

where *I*_0_ is the incident light intensity, *I* is the light intensity transmitted through the thin film, α is the absorption coefficient, and *d* is the film thickness. Since the absorbance is proportional to the BiVO_4_ film thickness, the fraction of the thickness that is etched during stability tests can be estimated by taking the ratio of the decreased and initial absorbance. Using the starting film thickness of approximately 60 nm and UV–vis measurements performed before and after stability testing, the etched thickness was then estimated.

### 2.7 Product Quantification

Product analysis was performed using an airtight two-compartment cell with the same configuration as the PEC measurements and with continuous stirring. After loading the cell, the system was flushed with He (Westfalen, 99.999%, for unbuffered conditions) or CO_2_ (Linde, 99.999%, for buffered conditions) for 45 min to remove air from the headspace. The valves on the cell were then closed to create an airtight system at room temperature and a pressure of ∼ 1 bar. Chronoamperometry was then performed, with the BiVO_4_ photoanode biased at 1.23 V_RHE_ and illuminated with simulated 1 Sun (AM1.5G) radiation. The pH values of the electrolytes were recorded using a Metrohm 781 pH meter, and gas and liquid samples were collected every 30 min.

The gas phase O_2_ and CO_2_ concentrations were analyzed using an SRI 8610C gas chromatograph (GC) equipped with a silica precolumn and molecular sieve 13X column coupled to a thermal conductivity detector (TCD, Amplifier 120 mA), using He (Westfalen, 99.999%) as carrier gas. To obtain precise values for O_2_ generated by the photoanode, it was important to account for the leakage of the PEC cell. Thus, after the initial step of flushing the system for 45 min, which resulted in O_2_ concentrations below the detection limit, blank experiments were performed in the sealed PEC cell without a photoanode loaded. During the experiments, O_2_ and N_2_ leaking into the system were measured over 2 h, as depicted in Figure S1a,b. Based on these measurements, the average TCD area ratio of oxygen (*A*_*O*_2__) to nitrogen (*A*_*N*_2__) was calculated to be approximately 0.41 ± 0.04. Based on this ratio, the oxygen produced only from the catalytic reaction (*A*_*O*_2__*reaction*_) was calculated as follows:

$$A_{O_{2\_\mathrm{reaction}}}=A_{O_{2}}-0.41A_{N_{2}}$$ 10

The concentrations of O_2_ and CO_2_ were quantified over the calibration range of 0.25–3.5% and 0.04–7%, respectively (Figure S1c). We note that air leakage was taken into account in the calibration curve of O_2_ by correction with the N_2_ signal.

For liquid phase product detection, H_2_O_2_ concentrations were measured via the colorimetric approach reported by Gill *et al.*,^3^ using an external standard prepared from known concentrations of H_2_O_2_ (Figure S1d). In brief, a 140 μL solution containing H_2_O_2_ (0–18 ppm, Sigma-Aldrich) was mixed with a 140 μL CoSO_4_ solution (0.07 M, Sigma-Aldrich, 99.99%) and 9.72 mL KHCO_3_ (0.5 M) electrolyte. After stirring for 30 min, the absorbance of the mixtures was measured. As expected from the Beer–Lambert Law, the H_2_O_2_ concentration was proportional to its absorption signal, enabling linear fitting to establish the calibration curve. To quantify the H_2_O_2_ generated by photoelectrochemical reactions, the colorimetric mixtures were prepared with the same approach, but using the collected electrolytes rather than the H_2_O_2_ standards.

Based on the detected concentrations of O_2_ and H_2_O_2_, Faradaic efficiencies (FEs) were calculated according to^44^

$$\mathrm{FE}=\frac{nFC}{Q}$$ 11

where *n* is the number of the holes required for generation of the product molecule (*n* = 4 for O_2_, *n* = 2 for H_2_O_2_), *F* is the Faraday constant, *C* is total molar quantity of the product generated, and *Q* is the total passed charge in the external circuit. The experiments were repeated three times to calculate the mean and error bars.

## 3 Results and Discussion

UV–vis optical absorption spectroscopy (Figure S2), X-ray diffraction (XRD, Figure S3), and scanning electron microscopy (SEM, inset Figure S3) measurements confirm successful fabrication of ∼ 60 nm thick monoclinic scheelite phase BiVO_4_ thin films with a bandgap of ∼ 2.5 eV. The physical properties of these thin films are, thus, in agreement with previous reports of active BiVO_4_ photoanodes.^40,45,46^

To explore the role of HCO_3_^–^ anions on water oxidation and photocorrosion mechanisms, PEC measurements were carried out in 0.5 M KPi buffer (pH 6.8), unbuffered 0.5 M KHCO_3_ electrolyte (pH 8.5), and buffered 0.5 M KHCO_3_ with continuous CO_2_ purging (pH 7.5), initially using an unsealed three electrode PEC cell. Although more concentrated (2 M) bicarbonate electrolytes have been shown to yield higher selectivities for HPER by inhibiting the subsequent photoanodic oxidation of H_2_O_2_ to O_2_, we chose less concentrated conditions to facilitate ICP-MS analysis of the BiVO_4_ corrosion process, as described later in this work.

Figure 1a–c shows the linear sweep voltammetry (LSV) measurements obtained with and without 1 M Na_2_SO_3_ hole scavenger in each of the three electrolytes. In the presence of hole scavenger, the photoelectrodes exhibit similar performance characteristics in all conditions. However, in the absence of hole scavenger, an approximately 2-fold larger photocurrent was observed in the bicarbonate-based electrolytes compared to in KPi buffer. Indeed, at anodic bias potentials near 1.23 V_RHE_, the current densities measured in both unbuffered and buffered KHCO_3_ approach the values measured in the same electrolytes with the hole scavenger present. To examine the possibility that the enhanced photocurrent arises from band bending induced by ion adsorption, experiments were performed using bicarbonate electrolytes containing different metal cations (Na^+^, K^+^, and Cs^+^). As demonstrated in Figure S4, the photocurrent responses were similar across the three bicarbonate electrolytes, indicating that the accompanying cation has a comparatively minor influence on the PEC performance. In contrast, measurements in pure KPi exhibit much smaller photocurrents that only gradually increase with increasing potentials.

**Figure 1:**
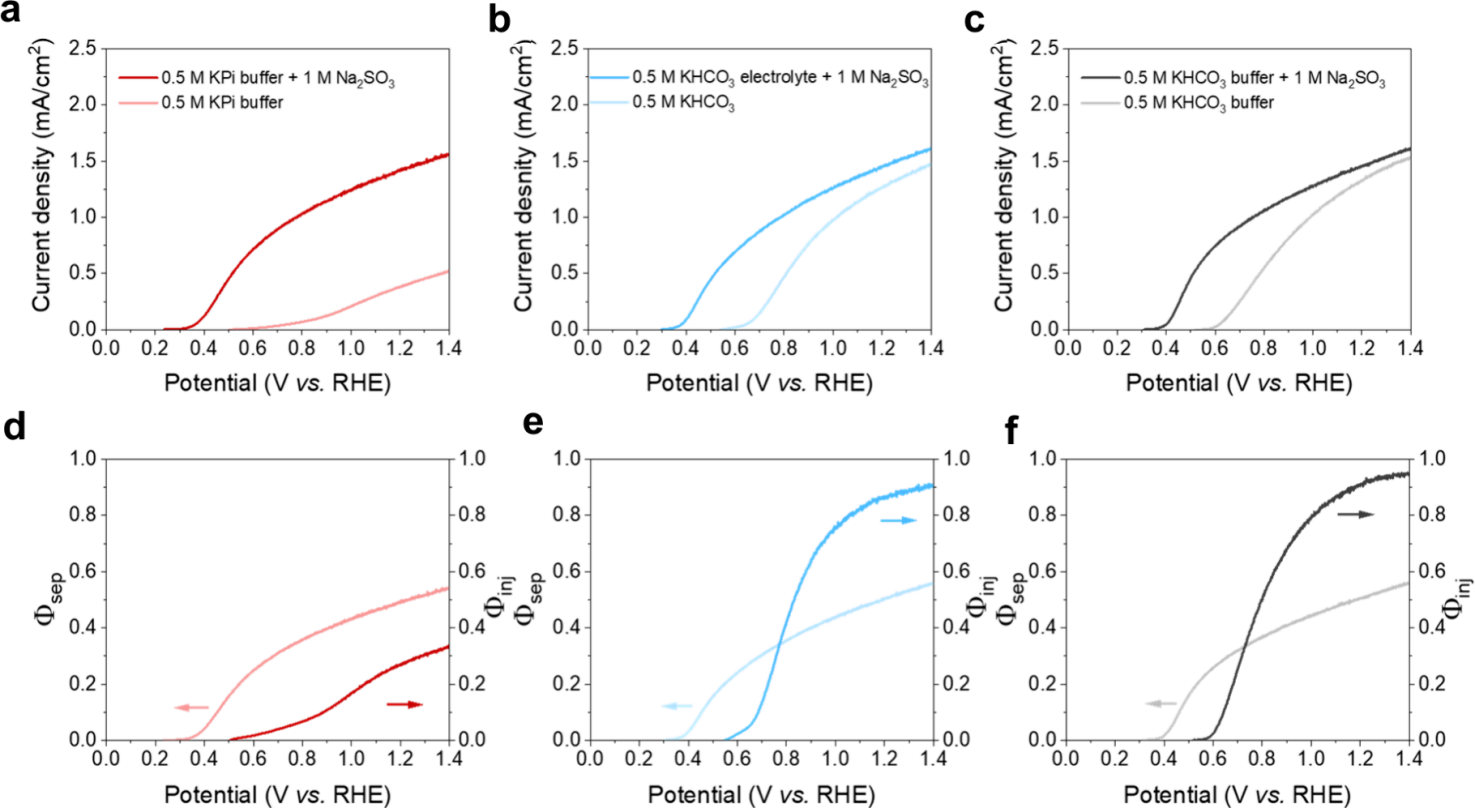
Linear sweep voltammetry (LSV) measurements of BiVO_4_ photoanodes recorded with and without 1 M Na_2_SO_3_ hole scavenger under 1 Sun illumination (100 mW/cm^2^, AM1.5G) in (a) 0.5 M KPi buffer, (b) 0.5 M unbuffered KHCO_3_ electrolyte, and (c) 0.5 M KHCO_3_ buffer under continuous CO_2_ purging with a flow rate of 50 sccm. The charge carrier injection and separation efficiencies, calculated according to eq 13, for (d) 0.5 M KPi buffer, (e) 0.5 M unbuffered KHCO_3_ electrolyte, and (f) 0.5 M KHCO_3_ buffer with CO_2_ purging.

To investigate the origin of the enhanced photocurrent densities in carbonate-containing electrolytes, we calculated the bulk charge separation efficiency (Φ_sep_) and the interfacial charge carrier injection efficiency (Φ_inj_), which together contribute to the measured photocurrent density, *J*, according to^41,42,47^

$$J=J_{\mathrm{abs}}\Phi_{\mathrm{sep}}\Phi_{\mathrm{inj}}$$ 12

where *J*_abs_ is the maximum saturation photocurrent density in the absence of recombination losses and is calculated to be 2.88 mA/cm^2^ based on the measured optical absorption characteristics of the BiVO_4_ thin films (eqs 4 **–** [Disp-formula eq7]). We note that this value is less than the theoretical limit for a 2.5 eV bandgap semiconductor, which is a consequence of the partial optical transparency of the 60 nm thick films used in this work. In the presence of the sacrificial hole acceptor, Φ_inj_ can be assumed to be close unity due to rapid sulfite oxidation. Therefore, Φ_sep_ and Φ_inj_ (Figure 1d–f) can be written as follows:

$$\Phi_{\mathrm{sep}}=\frac{J_{\mathrm{scavenger}}}{J_{\mathrm{abs}}}$$ 13a

$$\Phi_{\mathrm{inj}}=\frac{J_{\mathrm{no}\;\mathrm{scavenger}}}{J_{\mathrm{scavenger}}}$$ 13b

where *J*_scavenger_ and *J*_no scavenger_ are the photocurrent densities recorded in the presence and absence of Na_2_SO_3_, respectively.

Nearly identical values of Φ_sep_ (∼0.5 at 1.23 V_RHE_) are observed in each of the three types of electrolytes, which is consistent with all BiVO_4_ photoanodes possessing similar minority carrier diffusion lengths. Such a behavior is expected since the separation efficiency is primarily a bulk property of the semiconductor, and all electrodes were deposited under identical conditions. In contrast, the value of Φ_inj_ is highly dependent on the electrolyte, with measured values at an applied electrochemical potential of 1.23 V_RHE_ of 0.28 in KPi buffer, 0.88 in unbuffered KHCO_3_, and 0.93 in buffered KHCO_3_ buffer. Thus, we find that the presence of HCO_3_^–^ anions leads to a significant enhancement of the interfacial charge carrier injection efficiency. This observation provides evidence that HCO_3_^–^ ions play a direct role in the light-driven interfacial reaction mechanism. This effect is more pronounced in the CO_2_-purged KHCO_3_ buffer solution, which is likely a consequence of its greater ionic strength. In contrast, the comparatively low injection efficiency for the case of KPi buffer is consistent with prior reports and is commonly attributed to the kinetically sluggish OER,^48,49^ which leads to a less favorable competition with photocarrier recombination processes. Importantly, this poor OER activity of the bare BiVO_4_ electrode is a key characteristic underlying the ability of BiVO_4_ to selectively drive HPER.

In addition to changes in the magnitudes of the measured photocurrent densities in the three electrolyte environments, we observe key differences in their onset characteristics. In particular, the photocurrent densities and, thus, injection efficiencies, increase rapidly with increasing anodic potential, nearly reaching saturation for both the unbuffered and buffered KHCO_3_ electrolytes. By contrast, a much more gradual increase with potential is observed for the case of the buffered KPi electrolyte. Nevertheless, comparison of LSVs measured with and without hole scavenger reveal an anodic shift of the onset potential in all cases. For all electrolytes not containing hole scavenger, the photocurrent begins to onset at an approximately 300 mV more cathodic potential compared to when Na_2_SO_3_ is present. However, the observation of a comparatively steep photocurrent onset for the two bicarbonate-containing electrolytes suggests significantly accelerated oxidation kinetics compared to the phosphate electrolyte.

To provide additional insight into the influence of HCO_3_^–^ on the relative kinetics of interfacial hole injection and recombination, we performed transient photocurrent (TPC) measurements using a chopped 365 nm LED source (200 ms on/100 ms off). To ensure that dynamic responses were measured with excess photocarrier concentrations that are relevant to standard operating conditions, the photoelectrodes were simultaneously illuminated with a continuous simulated 1 Sun light bias during the chopped LED excitation. As shown in Figure 2, measurements in KPi buffer near the open circuit potential (*E*_oc_) result in significant photocurrent overshoots (undershoots) when the LED is switched on (off). Such transients are characteristic of recombination at the semiconductor/electrolyte interface. Importantly, the presence of HCO_3_^–^ leads to a near complete suppression of these transient characteristics, indicating that interfacial charge injection and the photoanodic oxidation reaction processes dominate over recombination under these conditions. In contrast, more pronounced transients are observed for the case of KPi buffer, consistent with the observation of lower photocurrent densities in LSV measurements that arise from the sluggish OER kinetics. Analogous outcomes were also obtained from TPC measurements performed solely under chopped LED illumination without a continuous light bias (Figure S5), with overshoots only observed for the case of the KPi buffer. Nevertheless, in all electrolytes, the magnitude of these transients gradually decreases as the applied potential increases, in agreement with the improved charge carrier separation efficiencies measured at more anodic bias potentials. Overall, both LSV and TPC measurements indicate faster photooxidation kinetics in the presence of HCO_3_^–^, suggesting that HCO_3_^–^ serves as either an active species within the catalytic cycle for water oxidation or as a hole scavenger,^50^ the details of which will be discussed below.

**Figure 2:**
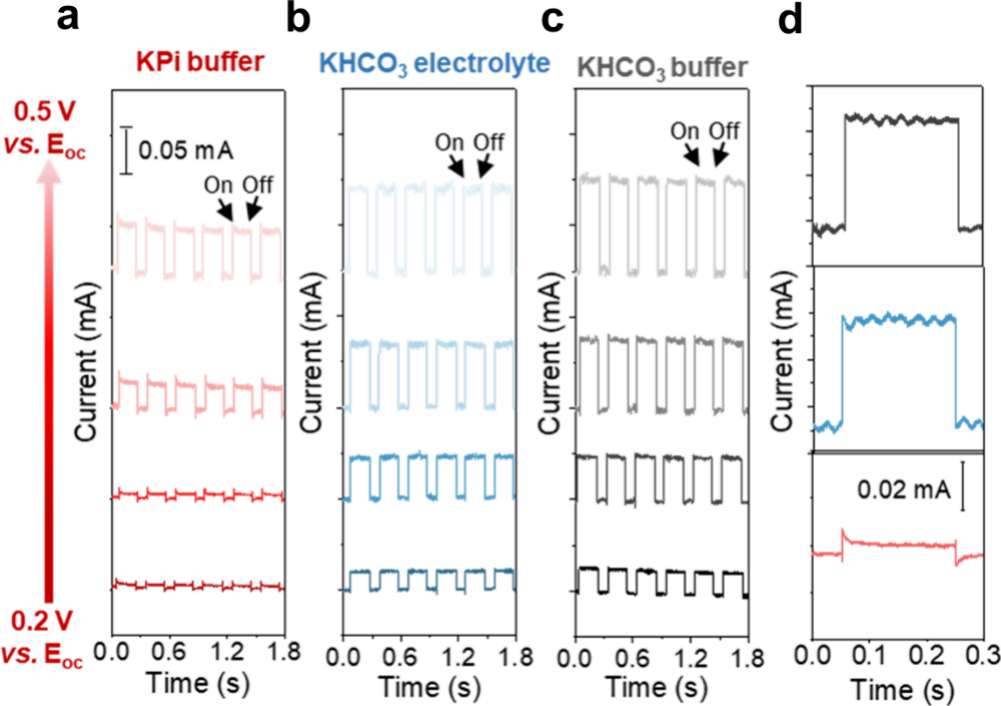
Transient photocurrent curves obtained in (a) 0.5 M KPi buffer, (b) 0.5 M unbuffered KHCO_3_ electrolyte, and (c) 0.5 M KHCO_3_ buffer with continuous CO_2_ purging. The data were collected during chopped illumination (200 ms on and 100 ms off times) with a 365 nm LED light source together with a continuous 1 Sun light bias from a solar simulator. (d) Zoomed in transients measured at 0.3 V vs *E*_*oc*_ in KPi buffer (red), KHCO_3_ electrolyte (blue), and KHCO_3_ buffer (black).

Before addressing the oxidation mechanisms occurring in different electrolytes, we next turn to the operational stabilities of the photoanodes. Previous studies have revealed that the accumulation of holes at the surface of BiVO_4_ can lead to chemical instabilities, resulting in significant photocorrosion. In this regard, the observation that the presence of HCO_3_^–^ leads to improved oxidation kinetics, along with reduced photocarrier recombination, implies that the bicarbonate anion may have a stabilizing influence on the material.^2^ Thus, to investigate the PEC stability of BiVO_4_ photoanodes, chronoamperometry (CA) tests were performed in each of the three electrolytes under 1 Sun illumination at an applied electrochemical potential of 1.23 V_RHE_ (Figure 3a–c). In agreement with prior observations, BiVO_4_ exhibits poor stability in KPi buffer, with the photocurrent density decreasing from ∼ 0.4 mA/cm^2^ to close to zero after 2 h of sustained operation. As noted above, this poor durability has been attributed to the sluggish kinetics of the four hole OER, which results in rapid photocorrosion.^27^ Here, this assignment is confirmed by the observation that photocorrosion can be suppressed in KPi buffer via the addition of hole scavenger to the electrolyte (Figure S6a,b). In contrast, much slower degradation is observed in the KHCO_3_ electrolyte, with the photocurrent density decreasing from ∼ 1.4 to 0.8 mA/cm^2^ during the course of the 2 h CA experiment, providing additional support for the finding that the presence of HCO_3_^–^ leads to more rapid extraction of photogenerated holes. Remarkably, photoanodic operation in the buffered bicarbonate electrolyte leads to a near complete suppression of photocorrosion, with stable photocurrent densities measured during the complete 2 h experiment, indicating that kinetic stabilization is highly effective under these conditions. As shown in Figure S6, similar stability differences between the three electrolytes are also observed during extended potential cycling.

**Figure 3:**
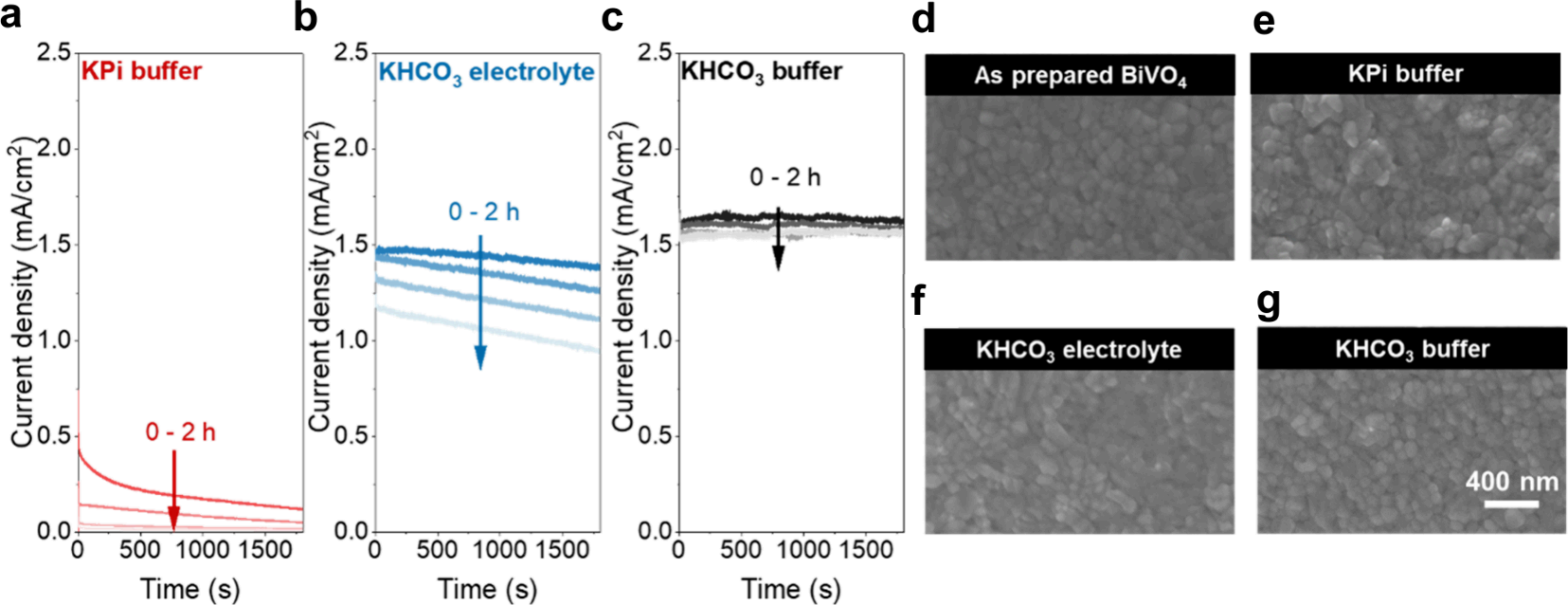
Chronoamperometry measurements on BiVO_4_ photoanodes, measured at 1.23 V_RHE_ with 1 Sun illumination in (a) 0.5 M KPi buffer, (b) 0.5 M unbuffered KHCO_3_ electrolyte, and (c) 0.5 M KHCO_3_ buffer, respectively. SEM top view images of (d) the as-prepared BiVO_4_ photoanode, along with BiVO_4_ photoanodes after 2 h chronoamperometry experiments in (e) 0.5 M KPi buffer, (f) 0.5 M unbuffered KHCO_3_, and (g) 0.5 M KHCO_3_ buffer. The scale bar is representative for all four micrographs.

To determine the influence of photocorrosion reactions on the morphology of the photoelectrodes, we compared plan-view SEM (Figure 3d–g) and AFM (Figure S7) images of the BiVO_4_ surfaces before and after the 2 h CA measurements. Before photoelectrochemical operation, the as-prepared BiVO_4_ thin films exhibited a compact morphology comprising small crystalline grains with an average size of ∼ 60 nm. Following 2 h of operation in KPi buffer at a bias of 1.23 V_RHE_, we observe a significantly roughened surface (R_q_ = 18.4 nm) and etched grains compared with as-prepared BiVO_4_ (R_q_ = 9.3 nm). This increased roughness is assigned to considerable corrosion that propagates from the free surfaces, consistent with previous findings.^27^ Weaker changes to the morphology and a slightly roughened surface (R_q_ = 11.2 nm) were observed following CA experiments in the unbuffered KHCO_3_ electrolyte, which is in agreement with its slower photocurrent degradation characteristics. In contrast, operation in buffered KHCO_3_ results in a negligible change of the surface morphology and minor roughening (R_q_ = 10.2 nm), confirming that photocorrosion is nearly completely suppressed under these conditions.

We note that the differences in the stability of BiVO_4_ photoanodes in unbuffered and buffered KHCO_3_ electrolytes is surprising, considering that the charge injection efficiency of the latter is only 5% larger than the former (Figure 1e,f). To understand this discrepancy, we consider that chemical, electrochemical, and photoelectrochemical instabilities can all be affected by the electrolyte environment, with dark processes potentially also contributing to the observed differences between unbuffered and buffered bicarbonate electrolytes. Therefore, we assessed the individual influences of illumination, applied bias, and pH using ICP-MS to determine the dissolved V and Bi ion concentrations in the electrolytes under various reaction environments. In particular, we measured the concentrations of these dissolved corrosion products in each of the three electrolytes under four conditions, with the electrodes held: (i) at the open circuit potential (*E*_oc_) in darkness (denoted as *E*_*oc*_^*dark*^), (ii) at 1.23 V_RHE_ in darkness (denoted as *E*_1.23 *V*_^*dark*^), (iii) at *E*_oc_ under 1 Sun illumination (denoted as *E*_*oc*_^*light*^), and (iv) at 1.23 V_RHE_ under 1 Sun illumination (denoted as *E*_1.23 *V*_^*light*^). Assuming uniform dissolution and using the measured dissolved Bi and V concentrations, together with the density of BiVO_4_ and the exposure time, eq 8 was then used to calculate the effective etching rate under each condition, as summarized in Figure 4 and Table S1. Under all conditions, we find that Bi and V are dissolved in nearly stoichiometric 1:1 ratios, thus corresponding to progressive etching rather than self-passivation by, for example, self-limited degradation to form a stable binary oxide on the surface. Complementary UV–vis absorption measurements performed before and after stability testing further confirm bulk dissolution, with corrosion-induced thinning leading to reductions of the optical density that can also be used to estimate the effective etching rate (Figure S8 and Table S1), resulting in trends that are in agreement with those obtained by ICP-MS.

**Figure 4:**
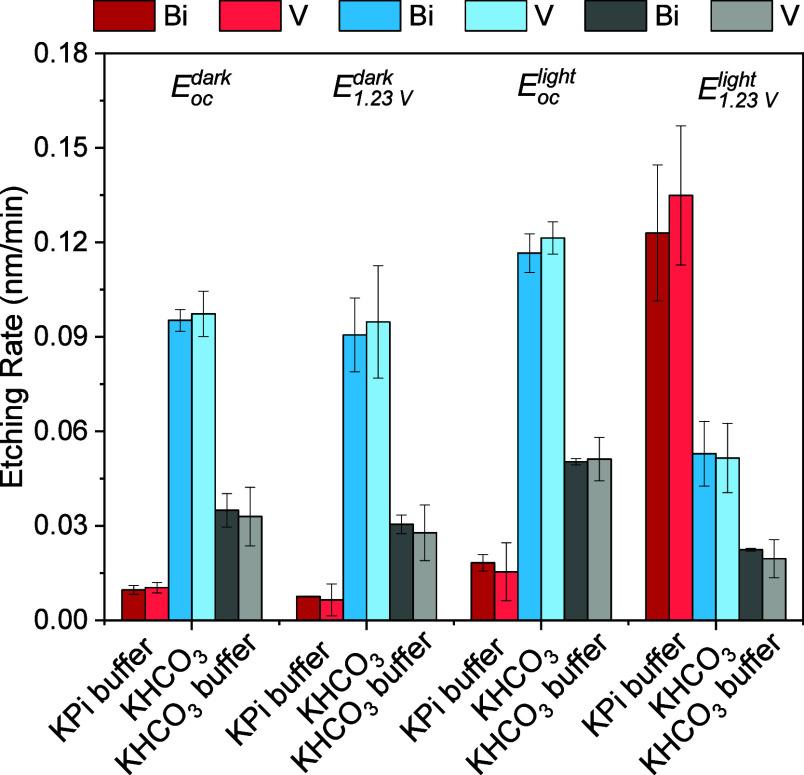
ICP-MS derived etching rates of BiVO_4_ photoanodes held at *E*_oc_ in darkness (*E*_*oc*_^*dark*^), at 1.23 V_RHE_ in darkness (*E*_1.23 *V*_^*dark*^), at *E*_oc_ under 1 Sun illumination (*E*_*oc*_^*light*^), and at 1.23 V_RHE_ under 1 Sun illumination (*E*_1.23 *V*_^*light*^) in each of the three different electrolytes.

Notable differences in stability are observed under the different investigated electrolyte, illumination, and bias conditions. Starting with the *E*_*oc*_^*dark*^ condition, we find a clear trend in the dark chemical etching rate as a function of electrolyte pH, with considerably larger rates observed in the pH 8.5 unbuffered KHCO_3_ electrolyte (0.1 nm/min), compared to the pH 7.5 buffered KHCO_3_ (0.03 nm/min) and the pH 6.8 buffered KPi (0.01 nm/min). Application of an applied bias in darkness (*E*_1.23 *V*_^*dark*^) does not result in any statistically significant changes in these etching rates. As a result of these dark measurements, we can thus conclude that the material is less chemically stable due to the more alkaline conditions in bicarbonate electrolytes, independent of the electrochemical bias condition.

In contrast to dark conditions, we find that illumination leads to pronounced bias-dependent changes in stability. While mild increases of the etching rate are observed under illumination at the open circuit potential (*E*_*oc*_^*light*^), much more significant changes occur at an applied bias of 1.23 V_RHE_ (*E*_1.23 *V*_^*light*^). For the case of KPi buffer, we observe a rapid etching rate of 0.13 nm/min, which represents an approximately order of magnitude enhancement. Moreover, degradation is nearly entirely suppressed when hole scavenger is added to the KPi buffer (Figure S6a,b). Thus, this process can be assigned to photocorrosion and is consistent with the previously proposed destabilization of BiVO_4_ via accumulation of photogenerated holes under the solid/liquid interface.^27^ Remarkably, this photocorrosion mechanism also appears to be nearly entirely suppressed in both unbuffered and buffered KHCO_3_, which can be attributed to the enhanced interfacial charge injection efficiency and, thus, reduced hole accumulation. Consistent with this assignment, addition of hole scavenger to the electrolyte results in negligible changes to the degradation rate for bicarbonate-containing electrolytes (Figure S6c–f). Moreover, for the case of unbuffered KHCO_3_ at *E*_1.23 *V*_^*light*^, we find a substantial reduction of the etching rate (0.05 nm/min) compared to all other bias and illumination conditions. By comparison, only a slight decrease in the etching rate (0.02 nm/min) is observed for the buffered KHCO_3_ under similar conditions.

Together, the above observations imply that pH changes at the surface of BiVO_4_ in the unbuffered KHCO_3_ electrolyte lead to reduced chemical etching rates compared to the dark and/or unbiased conditions. In particular, sustained photoanodic operation is expected to result in consumption of OH^–^, which leads to a reduction of the pH for the case of the unbuffered KHCO_3._ Although negligible changes in the bulk pH were measured in buffered KHCO_3_, we also observe an increase of the stability under biased illumination conditions. This behavior may indicate that consumption of OH^–^, together with mass transport and kinetic limitations to local equilibration, could also lead to a lower local pH at the photoanode/electrolyte interface in this case. Since the stability of BiVO_4_ is increased under less alkaline conditions, the photoanode is rendered more robust under operational conditions (*E*_1.23 *V*_^*light*^). Importantly, such stability differences imply that OH^–^ is consumed during the reaction process, as discussed in greater detail below.

Having established the impact of electrolyte environment on both interfacial charge injection and photoanode stability, we now turn to the role of HCO_3_^–^ on the light-driven oxidation mechanism. To this end, gas phase products (O_2_ and, when relevant, CO_2_) and liquid phase products (H_2_O_2_) were quantitatively analyzed using an airtight two-compartment PEC cell at room temperature and ambient pressure (see Supporting Information for details, and Figure S1 for calibration curves). To remove air from the headspace prior to the reaction, the cell was first flushed with He or CO_2_ for the unbuffered or buffered electrolyte, respectively. Following purging, gas and liquid products were sampled every 30 min during 2 h chronoamperometry experiments performed at an applied electrochemical potential of 1.23 V_RHE_ under 1 Sun illumination, corresponding to the *E*_1.23 *V*_^*light*^ condition described above.

Figure 5a shows the Faradaic efficiency (FE) for H_2_O_2_ and O_2_ in both unbuffered and buffered KHCO_3_ as a function of time during chronoamperometric testing, calculated according to eq 11 using the measured product concentrations (see Figure S9 for measured concentrations and Table S2 for specific values). Importantly, we observe considerable production of not only O_2_, but also H_2_O_2_, confirming that BiVO_4_ is active for HPER in electrolytes containing HCO_3_^–^. While total FEs of >90% are observed in both electrolytes and for all measurement times, we consistently observe higher overall FEs for the case of buffered compared to unbuffered KHCO_3_ electrolyte. However, closer inspection of the individual product contributions reveals statistically identical O_2_ FEs in both environments, whereas slightly increased H_2_O_2_ FEs are obtained in the buffered electrolyte. Here, it is important to note that H_2_O_2_ decomposition to O_2_ is increasingly favored under more alkaline conditions, such that the measured FE is not necessarily reflective of the absolute product branching ratio of the oxidation reaction. In addition, as previously noted, higher yields for H_2_O_2_ have been demonstrated in more concentrated bicarbonate-containing solutions, where the higher HCO_3_^–^ concentration favors HCO_4_^–^ generation over the kinetically competitive photoanodic hydrogen peroxide oxidation reaction (HPOR):

$$H_{2}O_{2}+4h^{+}\to O_{2}+2H^{+}$$ 14

**Figure 5:**
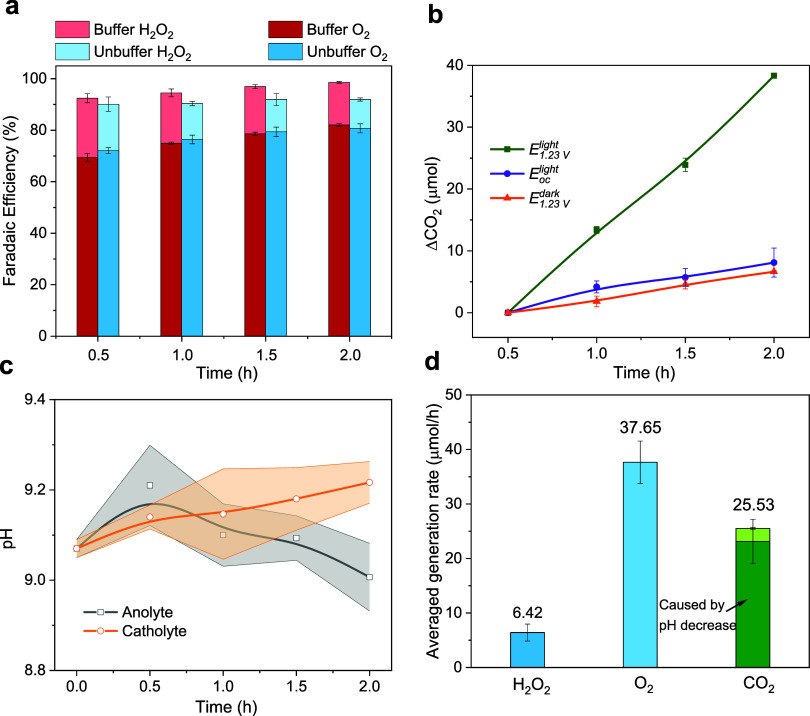
(a) Faradaic efficiencies for O_2_ and H_2_O_2_ generation during 2 h chronoamperometry measurements at 1.23 V_RHE_ under 1 Sun illumination (*E*_1.23 *V*_^*light*^) in buffered (red) and unbuffered (blue) 0.5 M KHCO_3_ electrolytes. (b) Increase of the gas phase CO_2_ amount, measured in the headspace of anolyte compartment, for the unbuffered KHCO_3_ electrolytes operated at *E*_1.23 *V*_^*dark*^, *E*_*oc*_^*light*^, and *E*_1.23 *V*_^*light*^. The concentration of CO_2_ is referenced to the value measured after 0.5 h to ensure stabilization of the system. (c) Measured pH change in the catholyte and anolyte compartments and (d) average generation rate of O_2_, H_2_O_2_, and CO_2_ under *E*_1.23 *V*_^*light*^ conditions in the unbuffered 0.5 M KHCO_3_ electrolyte between 0.5 and 2 h of the chronoamperometric experiment. The lines between data points in (b) and (c) are guides to the eyes, and the shaded regions in panel (c) represent the measurement uncertainty. In panel (d), the dark and light green regions indicate the CO_2_ evolution that can be attributed to the decrease of the anolyte pH and the PEC reaction, respectively.

Nevertheless, postcatalytic chemical or photochemical decomposition of H_2_O_2_ should not affect the overall FE to oxidation products since the net result is the release of O_2_. Thus, oxidation product analysis reveals that buffered conditions promote larger total FEs to O_2_ and H_2_O_2_, which could reflect the higher stability of the photoanode under these conditions.

Finally, to explore the stability of the electrolyte and to differentiate between the regenerative catalytic pathway (eq 2) and the nonregenerative scavenging pathway (eq 3), we next measured the CO_2_ concentration in the headspace during chronoamperometric testing in the unbuffered KHCO_3_ electrolyte under different bias and illumination conditions. To exclude contributions from CO_2_ produced by CO_2_/HCO_3_^–^ equilibration at the start of the experiment, the CO_2_ concentration in the headspace was referenced to the value measured after 0.5 h. Figure 5b shows the resulting increase of the amount of CO_2_ in the headspace, measured under conditions corresponding to *E*_*oc*_^*light*^, *E*_1.23*V*_^*dark*^, and *E*_1.23*V*_^*light*^. Importantly, we observe negligible increases of the CO_2_ concentration under both *E*_*oc*_^*light*^ and *E*_1.23*V*_^*dark*^. In contrast, the CO_2_ concentration continuously increases under *E*_1.23*V*_^*light*^, which is the only condition in which interfacial current is flowing.

While the observation of CO_2_ evolution under biased illumination conditions could suggest that HCO_3_^–^ decomposes via a hole scavenging pathway (eq 3), we also note that the photoanode stability tests described above implied a decrease of the anolyte pH during sustained operation under unbuffered conditions. The pH affects the carbonate equilibrium and CO_2_ production (as described by the Bjerrum diagram), according to

$${\mathrm{CO}}_{2}+H_{2}O⇌{{\mathrm{HCO}}_{3}}^{-}+H^{+}$$ 15a

$${{\mathrm{HCO}}_{3}}^{-}⇌{{\mathrm{CO}}_{3}}^{2-}+H^{+}$$ 15b

Therefore, we monitored the pH of both the anolyte and catholyte during the CA experiment. As shown in Figure 5c, the pH of the catholyte continuously increased during the experiment, which is expected as the hydrogen evolution reaction (HER) proceeds under unbuffered conditions. In contrast, we observe an initial increase and then a steady decrease of the pH in the anolyte. Furthermore, we note that the initial pH of the KHCO_3_ electrolyte is higher in the airtight than in the open system (Table S1). Both this initial rise and higher starting pH can be attributed to degassing of the electrolyte by He purging, which results in an initial decrease of the dissolved and gas phase CO_2_ concentration and, thus, an increase in the pH according to eq 15. Reference measurements of the CO_2_ concentration in the headspace of a purged cell but in the absence of electrical bias (*i.e.*, without a photoanodic reaction), indicate that CO_2_/HCO_3_^–^ equilibrium is established within the first 0.5 h of operation (Figure S10), such that further changes can be attributed to the action of the photoanode. Importantly, following this initial 0.5 h stabilization time, the anolyte pH steadily decreases, which indicates photoanodic consumption of OH^–^, as expected for both HPER and OER.

We next sought to determine whether the evolved CO_2_ is driven by the observed change of the pH or if it is a direct consequence of a hole scavenging photoanodic reaction pathway. For this, we consider the change of the CO_2_ partial pressure in the headspace due to the dissolved CO_2_ concentration between the end of the 2 h experiment (pH 9.0) and after initial stabilization for 0.5 h (pH 9.2), according to Henry’s law:

$${\mathrm{\Delta p}}_{{\mathrm{CO}}_{2}}=k\cdot \Delta \left[\mathrm{CO}2\right]=k\left({\left[\mathrm{CO}2\right]}_{\mathrm{pH}9}-{\left[\mathrm{CO}2\right]}_{\mathrm{pH}9.2}\right)$$ 16

where k is the Henry constant (29.4 L atm/mol). The pH-dependent CO_2_ concentration in eq 16 can be estimated as^51^

$$\left[{\mathrm{CO}}_{2}\right]=DIC\frac{{\left[H^{+}\right]}^{2}}{{\left[H^{+}\right]}^{2}+\left[H^{+}\right]K_{1}+K_{1}K_{2}}$$ 17

where *DIC* is the total inorganic carbon concentration (0.5 mol/L), and K_1_ (4.5 × 10^–7^ mol/L) and K_2_ (4.8 × 10^–11^ mol/L) are the dissociation constants of the reactions given by eqs 15a and 15b, respectively. Based on the change of partial pressure, the pH-induced CO_2_ production rate (*ṅ*_CO_2__) can be calculated using the ideal gas law and the reaction time, *t*, as follows:

$${ṅ}_{{\mathrm{CO}}_{2}}=\frac{V_{\mathrm{headspace}}\Delta_{p_{{\mathrm{CO}}_{2}}}}{RT}\frac{1}{t}$$ 18

where *V*_*headspace*_ is the headspace volume, *R* is the ideal gas constant, and *T* is the room temperature.

Figure 5d illustrates the calculated production rates during the final 1.5 h of the 2 h CA tests. On the basis of these calculations, we find that the CO_2_ production rate due to the decreased pH that is induced by the combination of HPER and OER under unbuffered electrolyte conditions is 23.13 ± 4.05 μmol/h. Considering the experimental uncertainties, this calculated rate is statistically indistinguishable from the measured rate (25.53 ± 0.18 μmol/h), suggesting that effectively all HCO_3_^–^ anions can be regenerated. Although the observed reaction-induced CO_2_ evolution is negligible, it is important to note that unbuffered conditions do lead to a change of anolyte pH that can lead to the irreversible loss of CO_2_ in open systems. Combined with the reduced stability of BiVO_4_ under unbuffered alkaline conditions, buffered bicarbonate-containing electrolytes can enable more robust operation by suppressing degradation of the photoelectrode and providing a more stable catalytic environment.

## 4 Conclusions

In summary, we find that bicarbonate-based electrolytes dramatically improve the performance of BiVO_4_ photoanodes relative to bicarbonate-free electrolytes, with HCO_3_^–^ ions serving as highly efficient mediators that rapidly extract photogenerated holes and nearly completely eliminate interfacial recombination. As a consequence, photocorrosion rates are significantly suppressed in bicarbonate-containing electrolytes. Nevertheless, comparison of BiVO_4_ stability under different electrolytes, bias, and illumination conditions via ICP-MS reveals that BiVO_4_ is susceptible to pH-dependent chemical attack, with near-neutral buffered KHCO_3_ solutions being significantly more stable than mildly alkaline unbuffered electrolytes. In addition, quantitative product analysis confirms that HCO_3_^–^ anions can regeneratively mediate both HPER and OER. However, continuous operation in unbuffered KHCO_3_ leads to a decrease of the pH of the anolyte, resulting in evolution of CO_2_. For the case of open reactor configurations, such a process could lead to an irreversible electrolyte loss and would have a considerable influence on the overall energy balance for HPER. Thus, we find that operation of BiVO_4_ photoanodes in buffered bicarbonate-containing electrolytes not only enables sustainable generation of high-value H_2_O_2_ but also maintains the chemical and photochemical stability required for integrated solar chemical generators. Indeed, such electrolyte conditions make these photoanodes directly compatible with coupled (photo)­cathodic CO_2_ reduction systems that can simultaneously provide high value oxidation and reduction products. Overall, these results highlight the critical role of electrolyte engineering for overcoming the inherent instabilities of BiVO_4_ photoanodes, providing a route to more durable and efficient solar-to-chemical energy conversion systems. Moreover, these insights motivate future investigations of a broader range of other photoanode materials to determine whether bicarbonate anion-mediated reactions can improve stability and efficiency, while also enabling H_2_O_2_ generation as a valuable oxidation product.

## Supplementary Material



## Notes

Optical, morphological and structural characterization, and product calibration and quantification, including O_2_, H_2_O_2_ and CO_2_ (PDF)
