# Supplementary material for: Water Oxidation and Degradation Mechanisms of BiVO4 Photoanodes in Bicarbonate Electrolytes
Source: ACS Catal. 2025 Jul 16;15(15):13048–58. doi: 10.1021/acscatal.5c03025 (PMC12322915; doi:10.1021/acscatal.5c03025)
Supplement: Supplementary file 1 [file cs5c03025_si_001.pdf]

Supporting Information

Water Oxidation and Degradation Mechanisms of $BiVO_4$ Photoanodes in Bicarbonate Electrolytes

Guanda Zhou,[a,b] Clara C. Aletsee,[c] Anna Lemperle,[c] Tim Rieth,[a,b] Lucia Mengel,[c] Jianyong Gao,[a,b] Martin Tschurl,[c] Ueli Heiz,[c] Ian D. Sharp,[a,b,]*

[a] Walter Schottky Institute, Technical University of Munich, 85748 Garching, Germany
[b] Physics Department, TUM School of Natural Sciences, Technical University of Munich, 85748 Garching, Germany
[c] Chair of Physical Chemistry, TUM School of Natural Sciences and Catalysis Research Center, Technical University of Munich, 85748 Garching, Germany

*Corresponding author e-mail: sharp@wsi.tum.de

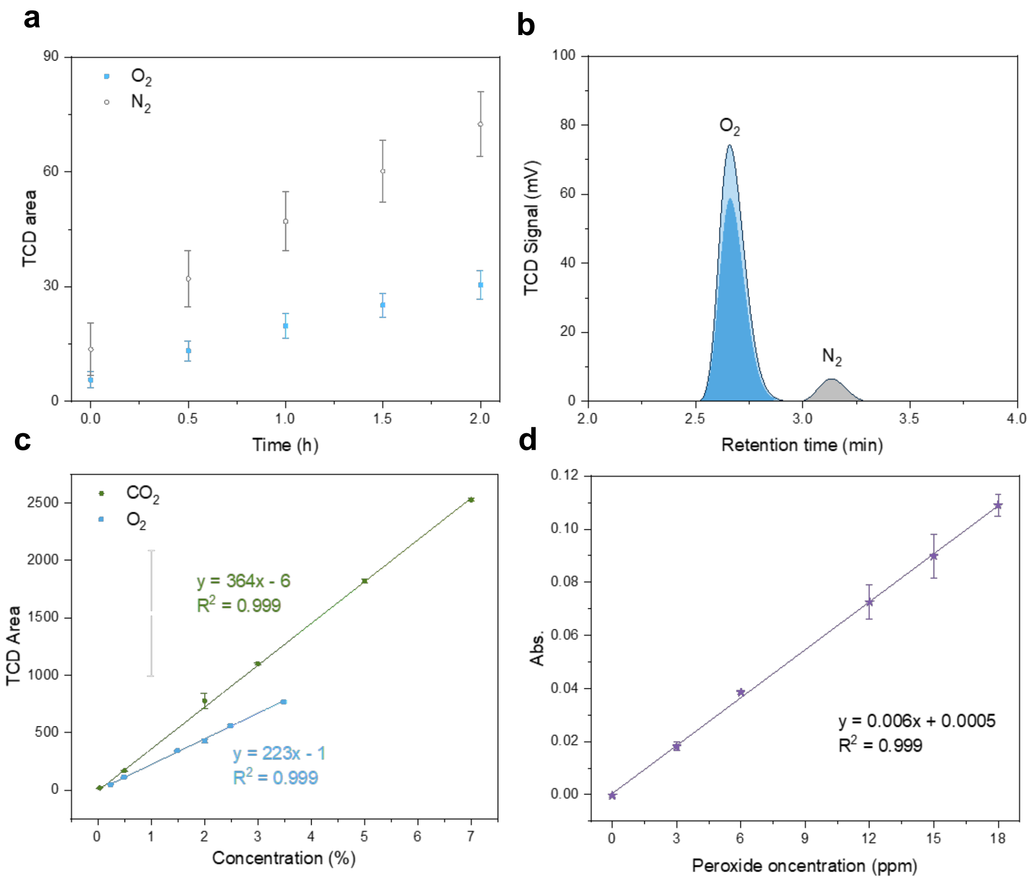

Figure S1. (a) TCD area of leaked O_2 and N_2 into the anolyte compartment during 2 h blank experiment without $BiVO_4$ photoanodes. (b) Depiction of the O_2 correction procedure, where the dark-blue area represents the O_2 amount produced in the reaction, whereas the light-blue area arises from air leakage into the anolyte compartment, which was quantified by the TCD area of N_2 (gray area). (c) Calibration curves of O_2 and CO_2 obtained by TCD detection with He as carrier gas. (d) Calibration curve of H_2O_2 using colorimetry.

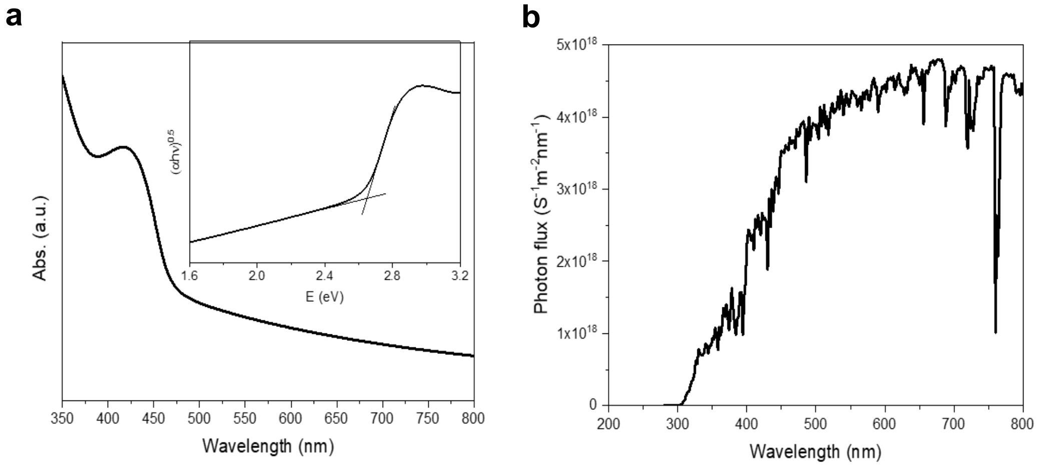

Figure S2. (a) UV-Vis absorption spectrum of as-prepared $BiVO_4$ on a fused silica substrate and (insert) the corresponding Tauc plot for an indirect bandgap semiconductor. (b) ASTM G173 AM1.5G solar spectrum used for calculation of J_{abs}.[1]

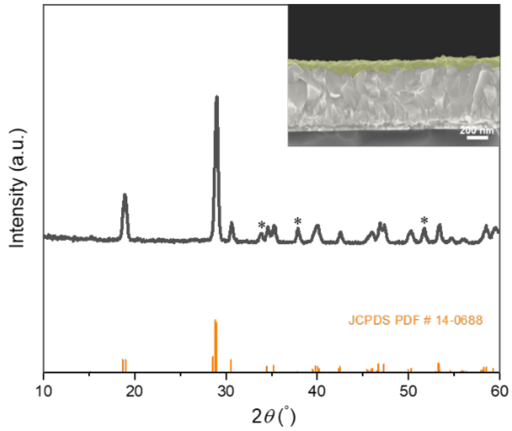

Figure S3. XRD pattern of fabricated $BiVO_4$ on FTO substrate (black), and the standard XRD pattern of monoclinic scheelite $BiVO_4$ (orange). Diffraction peaks arising from the FTO substrate are marked with *. The insert shows a cross-sectional SEM image of the as-prepared $BiVO_4$ on FTO, with the yellow color added to indicate the location of the film.

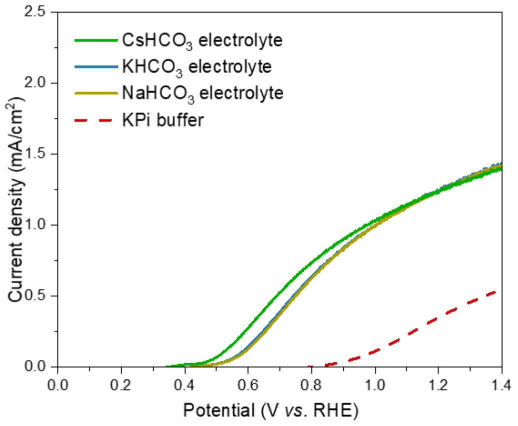

Figure S4. Linear sweep voltammetry measurements in 0.5 M bicarbonate electrolytes comprising different metal cations (K^+, Na^+ and Cs^+), measured under 1 Sun conditions (100 mW/cm^2, AM1.5G).

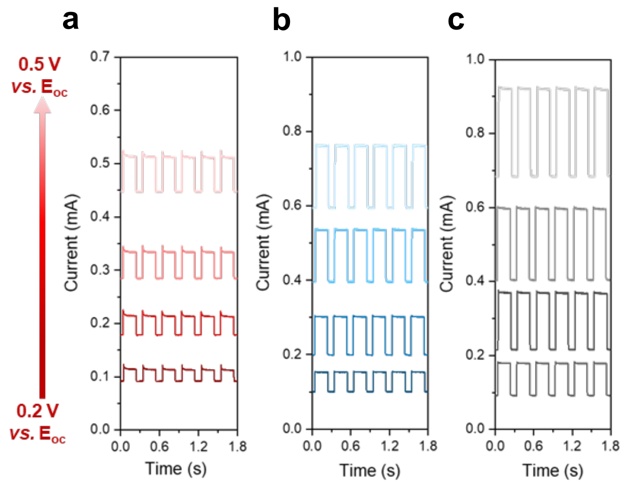

Figure S5. Transient photocurrent measurement at specific potentials under 365 nm chopped LED illumination (200 ms on and 100 ms off) at different applied electrochemical potentials in (a) 0.5 M KPi buffer, (b) 0.5 M unbuffered $KHCO_3$ electrolyte, and (c) 0.5 M $KHCO_3$ buffer with continuous CO_2 bubbling.

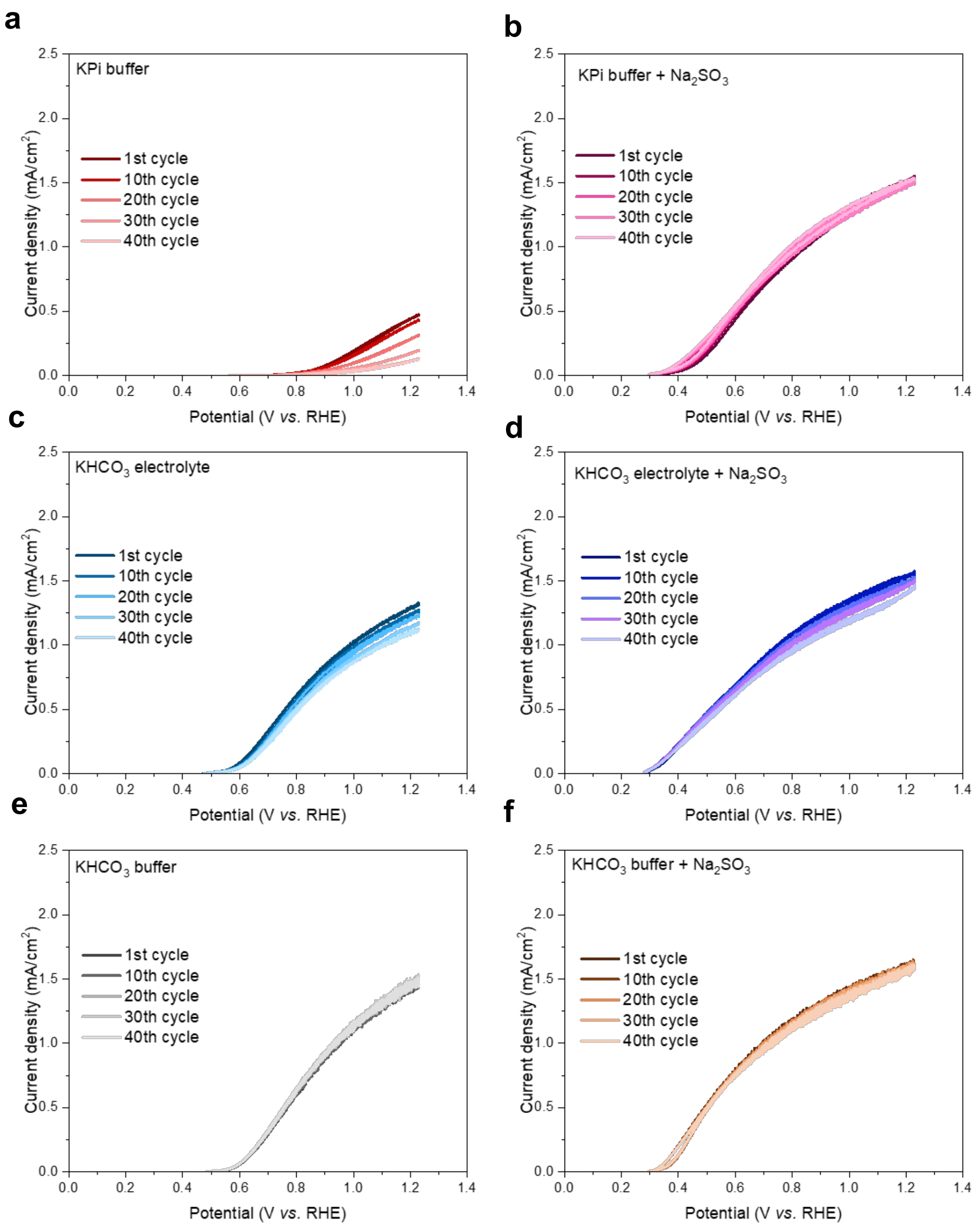

Figure S6. Extended cyclic voltammetry stability measurements (40 cycles) performed in (a) 0.5 M KPi buffer, (b) 0.5 M KPi buffer with Na_2SO_3, (c) 0.5 M $KHCO_3$ electrolyte, (d) 0.5 M $KHCO_3$ electrolyte with Na_2SO_3, (e) 0.5 M $KHCO_3$ buffer, and (f) 0.5 M $KHCO_3$ buffer with Na_2SO_3. All measurements were performed under 1 sun illumination conditions (100 mW/cm^2, AM1.5G).

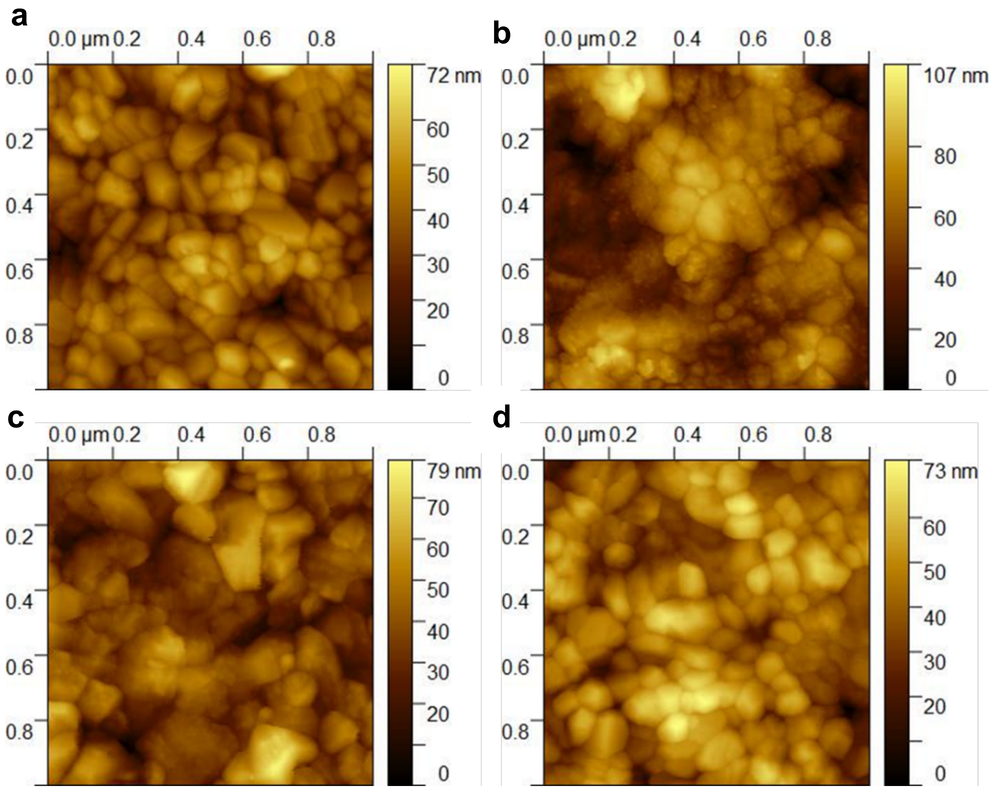

Figure S7. AFM images used to evaluate morphological changes and determine rms roughness values for (a) the as-prepared $BiVO_4$ photoanode (R_q = 9.3 nm), as well as $BiVO_4$ photoanodes after 2 h chronoamperometry experiments in (b) 0.5 M KPi buffer (R_q = 18.4 nm), (c) 0.5 M unbuffered $KHCO_3$ (R_q = 11.2 nm), and (d) 0.5 M $KHCO_3$ buffer (R_q = 10.2 nm).

Table S1. Stability of $BiVO_4$ in Different Electrolytes

Electrolyte	Illumination	Potential (V_{RHE})	Anolyte pH before reaction	Anolyte pH after reaction	Degradation rate based on Bi determined by ICP-MS (nm/min)	Degradation rate based on V determined by ICP-MS (nm/min)	Degradation rate determined by UV-Vis (nm/min)
KPi buffer	off	0	6.85	6.88	0.01 ± 0.001	0.01 ± 0.002	0.02 ± 0.006
	off	1.23	6,84	6.84	0.01 ± 0.001	0.01 ± 0.005	0.03 ± 0.016
	on	0	6.83	6.82	0.02 ± 0.003	0.02 ± 0.009	0.06 ± 0.028
	on	1.23	6.86	6.84	0.12 ± 0.022	0.13 ± 0.022	0.14 ± 0.012
$KHCO_3$ electrolyte	off	0	8.5	8.76	0.10 ± 0.003	0.10 ± 0.007	0.08 ± 0.011
	off	1.23	8.47	8.74	0.09 ± 0.012	0.09 ± 0.018	0.08 ± 0.041
	on	0	8.41	8.75	0.12 ± 0.006	0.12 ± 0.005	0.13 ± 0.030
	on	1.23	8.46	8.64	0.05 ± 0.010	0.05 ± 0.011	0.07 ± 0.039
$KHCO_3$ buffer	off	0	7.51	7.53	0.03 ± 0.005	0.03 ± 0.009	0.06 ± 0.018
	off	1.23	7.44	7.47	0.03 ± 0.003	0.03 ± 0.009	0.05 ± 0.010
	on	0	7.39	7.41	0.05 ± 0.001	0.05 ± 0.007	0.04 ± 0.015
	on	1.23	7.43	7.41	0.02 ± 0.001	0.02 ± 0.006	0.03 ± 0.007

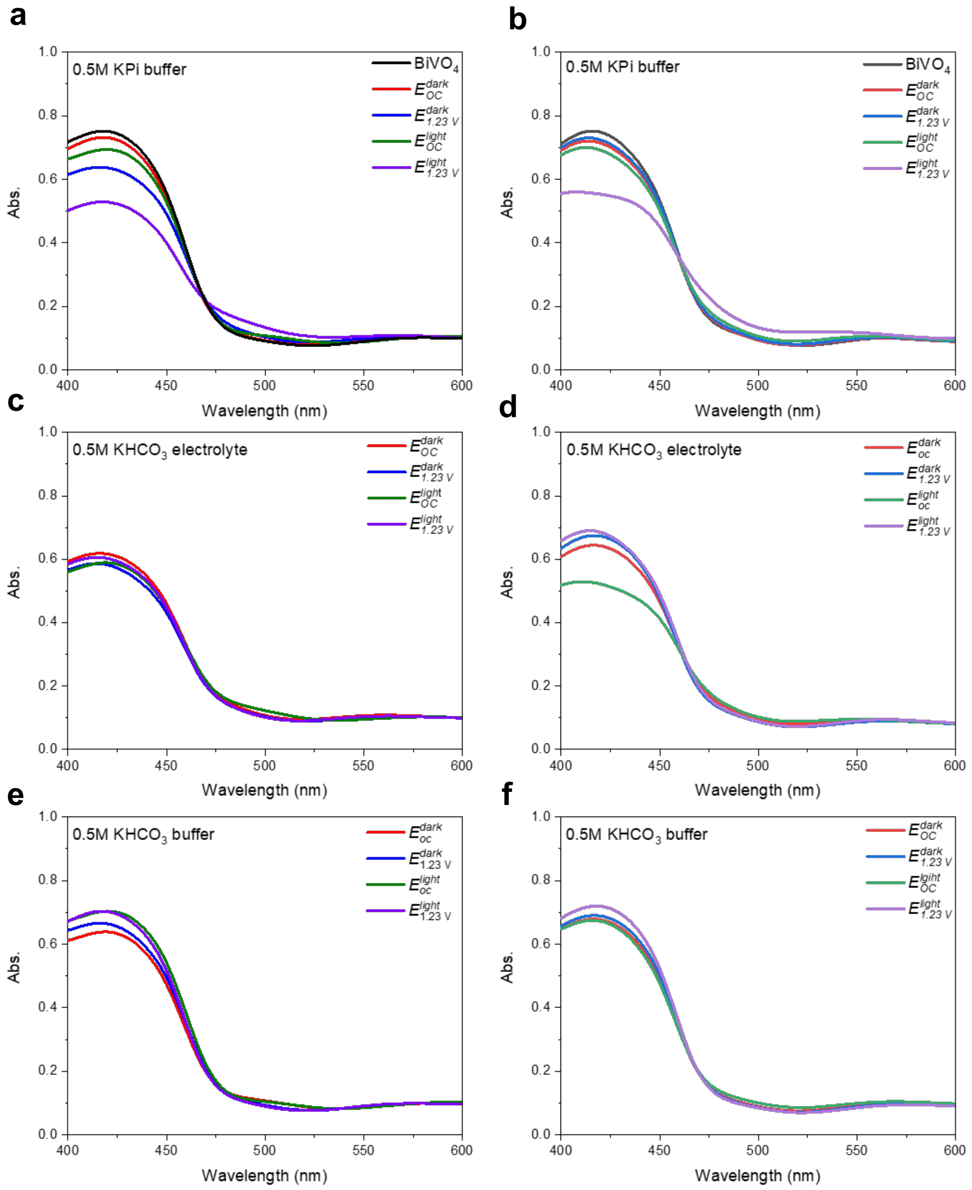

Figure S8. UV-Vis spectra of $BiVO_4$ photoanodes on FTO substrates before and after reaction under different conditions in (a) and (b) 0.5 M KPi buffer, (c) and (d) 0.5 M unbuffered $KHCO_3$ electrolytes, and (d) and (e) 0.5M $KHCO_3$ buffer. All experiments were performed twice, with results from each presented in the data given in the left and right columns.

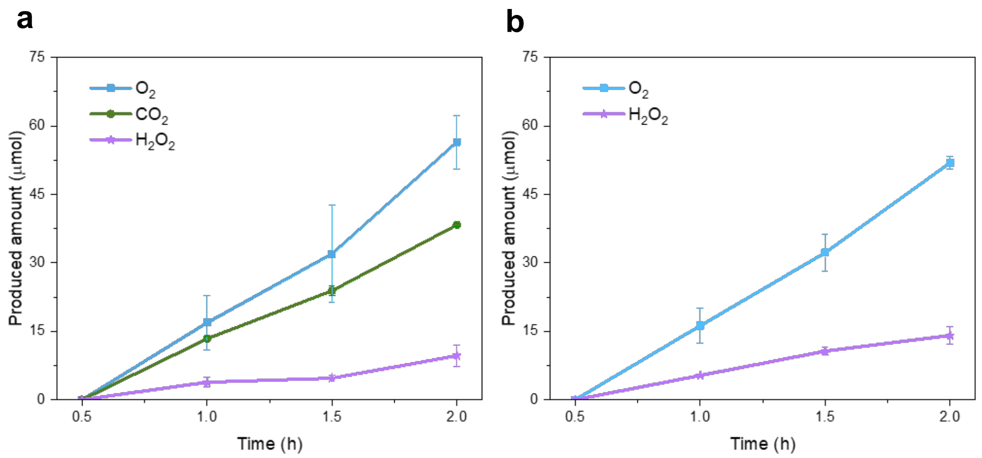

Figure S9. Time-dependent product evolution, referenced to the results obtained after a stabilization time of 0.5 h in (a) 0.5 M unbuffered $KHCO_3$ electrolyte and (b) 0.5 M $KHCO_3$ buffer. The concentrations of CO_2 and O_2 were determined by GC and the values for H_2O_2 were ascertained by the colorimetric method. The lines between data points are guides to the eyes.

Table S2. Summary of Faradaic Efficiencies in Different Electrolyte

Time (h)	Product	$KHCO_3$ electrolyte	$KHCO_3$ buffer
	O_2	72.06 ± 1.18	69.43 ± 1.53
0.5	H_2O_2	18.03 ± 2.84	23.01 ± 1.75
	O_2	76.42 ± 1.69	74.95 ± 0.43
1	H_2O_2	13.91 ± 0.84	19.55 ± 1.53
	O_2	79.39 ± 1.84	78.61 ± 0.66
1.5	H_2O_2	12.54 ± 2.36	18.33 ± 0.80
	O_2	80.74 ± 1.75	82.03 ± 0.51
2	H_2O_2	11.18 ± 0.61	16.55 ± 0.37

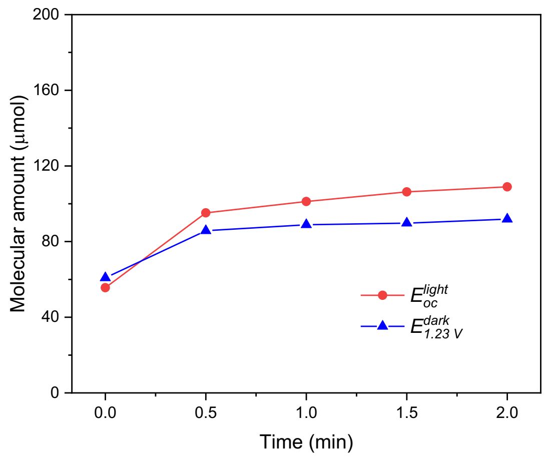

Figure S10. The molecular amount of CO_2 in the headspace of the catholyte compartment during 2 h experiments in He-purged unbuffered 0.5 M $KHCO_3$ under applied bias only ($E_{1.23\ V}^{dark}$, blue) and illumination only (E_{oc}^{light}, red). No current through the working electrode is observed under these conditions and the CO_2 level plateaued after 0.5 h, indicating that the CO_2/HCO_3^- equilibrium has been reached. The lines between data points are guides to the eyes.

References

(1) G173 AM 1.5G spectrum. *The American Society for Testing and Materials* **2003**.
